# Assessing Pediatric Dental Stress Through Wearable Technology: Influence of Procedure Type, Treatment Phase, and Age

**DOI:** 10.1055/s-0045-1813651

**Published:** 2025-12-23

**Authors:** Francesco Saverio Ludovichetti, Julia Gonçalves de Freitas, Claudia Manera, Patrizia Lucchi, Federica I. Giordano, Edoardo Stellini, Sergio Mazzoleni

**Affiliations:** 1 Neurosciences Department, Dentistry Section, Università Degli Studi di Padova, Padova, Italy

**Keywords:** pediatric dentistry, heart rate variability, stress assessment, behavioral management

## Abstract

**Objective:**

Pediatric dental stress is a common barrier to effective treatment, often resulting in behavioral management challenges and long-term avoidance of dental care. Wearable technology, such as smart watches capable of measuring heart rate variability (HRV), may offer real-time, noninvasive tools to assess stress in clinical settings. This study aimed to evaluate physiological stress responses in children undergoing different dental procedures by analyzing HRV across treatment phases.

**Materials and Methods:**

Eighty children aged 5 to 10 years (mean age = 7.2 ± 1.6 years) were randomly assigned to four treatment groups: dental hygiene, dental restorations with anesthesia, restorations without anesthesia, and sealant application. Stress levels were measured using Garmin smart watches that analyze HRV and convert the data into a stress score from 0 to 100. Stress was recorded at three time points—beginning, during, and end of the procedure—for a total of nine measurements per patient. A two-way repeated measures ANOVA was used to evaluate the effects of treatment type and phase, along with post hoc tests and linear regression to assess age-related stress variation.

**Results:**

While the type of treatment alone did not significantly influence stress levels, stress varied significantly across treatment phases ( *p* = 0.0249), with a strong interaction between treatment and phase ( *p* = 0.0004). Post hoc analyses revealed that dental hygiene led to a significant reduction in stress over time ( *p* < 0.05), whereas restorations with anesthesia caused a significant increase in stress during the procedure ( *p* = 0.0011). No significant changes were observed for restorations without anesthesia and sealant application. Age was inversely correlated with stress ( *p* = 0.0003), although it explained only a small proportion of variance ( *R* ^2^ = 0.0527).

**Conclusion:**

The study confirms that pediatric dental stress is influenced by both the procedure type and the treatment phase. Smart watches represent a practical tool for monitoring stress in real time. These findings highlight the importance of tailoring behavior management strategies to the procedure and the patient's age, promoting individualized and stress-reducing approaches in pediatric dental care. From a clinical perspective, wearable stress monitoring can assist pediatric dentists in real-time identification of anxiety peaks, allowing timely behavioral adjustments and potentially improving treatment outcomes and patient cooperation.

## Introduction

Pediatric dentistry is a recognized dental specialty dedicated to the oral health care of infants, children, and adolescents, including individuals with special health care needs. It provides both primary and comprehensive preventive and therapeutic services. 1 As a specialized field, it requires specific expertise and skills from dentists due to the unique health and psychological needs of children. It plays a fundamental role in providing healthy oral habits that can last a lifetime. 2 Oral health is important to the general wellness of infants, children, and adolescents, as oral diseases can affect function, development, and life quality. 3

Treating children can be a highly rewarding experience for a dentist. When approached with the right mindset, training, and environment, it offers an enjoyable experience for both the child and the practitioner. 4 However, pediatric dentistry presents a range of challenges.

Early childhood caries continues to be considered the most common chronic disease among children in the United States, impacting 60 to 90% of school-age children. 5 Dentists can treat caries by using rotary instruments, not local anesthesia, followed by filling the cavity. 6 Early interventions by dental professionals can significantly reduce the risk of dental caries, including the application of fissure sealants and fluoride varnish treatments. 7

Dental sealants, also known as pit and fissure sealants, are protective coatings made by plastic layers that are applied to the chewing surfaces of teeth to protect deep grooves and depressions, which are common sites for cavities in children and teenagers. 8 9 10 activity of certain bacterial enzymes. 10

Epidemiological studies have shown that dental plaque and its biofilm are the primary cause of the most common form of gingivitis in children and adolescents. 11 Effective daily oral hygiene practices, combined with regular professional periodontal care, are critical in controlling plaque accumulation and maintaining gingival health. 11 12 13

Preventive strategies have made significant progress, but pediatric dentists still face challenges with children's fear, anxiety, and stress, which can affect the efficacy of treatment. 14

Dental fear is considered a normal but unpleasant emotional response to specific stimuli in dentistry, whereas dental anxiety is an exaggerated and irrational negative emotional state experienced by patients. 15 Children with dental fear often show poor cooperation and frequently try to avoid or delay treatment, which worsens their oral health. 16 Stress is a natural and common experience that every human will go through. While it often has beneficial effects, prolonged or excessive stress can negatively impact the body. Stress levels are divided from “normal” to “extremely severe” based on percentile scores: 0 to 78 is deemed to be “normal,” 78 to 87 is “mild,” 87 to 95 is “moderate,” 95 to 98 is “severe,” and 98 to 100 becomes “extremely severe.” 17

Over the past decade, wearable technology, particularly smart watches, has gained significant popularity. Smart watches have evolved to address broader aspects of health, including stress management.

One such feature is heart rate variability (HRV) analysis. HRV refers to the small changes in the time between heartbeats. It's usually calculated with an ECG or special sensors, and it is used as a metric to evaluate the functioning of the autonomic nervous system (ANS). The ANS, which regulates involuntary bodily functions such as heart rate and respiration, is divided into two systems. The sympathetic nervous system (SNS) oversees the fight or flight response, and the parasympathetic nervous system, which is responsible for the rest and digest functions.

HRV reflects the dynamic balance between these two branches. A high HRV suggests strong parasympathetic activity and greater adaptability to stress, while a low HRV indicates sympathetic dominance, which could mean chronic stress, exhaustion, or other health problems.

Nowadays, smart watches are capable of tracking heart rate and HRV. Since stress and anxiety are associated with an elevated heart rate and reduced HRV, real-time analysis of these metrics allows for the identification of stress patterns and potential triggers. Moreover, HRV analysis provides insight into the balance of the ANS, making these devices useful for self-regulating stress and improving overall health. 18

HRV analysis through wearable devices offers an innovative, noninvasive way to monitor stress in real time. By quantifying ANS activity, HRV enables the objective assessment of physiological stress responses during dental procedures (DP). 17 18 Despite its potential, limited evidence exists regarding how HRV-based stress scores vary according to the type and phase of pediatric dental treatments.

Traditional HRV assessment typically relies on electrocardiography or specialized laboratory instruments that, while highly accurate, are impractical for continuous monitoring in dental settings—especially with young children who may find wires and electrodes uncomfortable or intimidating. Wearable technology overcomes these limitations by providing real-time, contactless data acquisition through optical sensors integrated into smartwatches. Previous validation studies have shown a strong correlation between smartwatch-derived HRV and ECG-based measurements, supporting their reliability for stress assessment in both adults and children. Therefore, using a wearable device in this study allowed for a realistic, noninvasive evaluation of physiological stress directly during dental care, enhancing the ecological validity of the data while ensuring patient comfort.

The aim of this study is to measure stress in pediatric patients undergoing various DP, such as professional dental hygiene, dental sealant application, and dental restoration with and without the use of anesthesia, by analyzing HRV as a physiological marker of stress.

Based on these premises, the study aimed to determine whether stress levels in pediatric patients differ according to the type and phase of dental treatment, and whether age plays a role in modulating these responses. It was hypothesized that stress would vary significantly across treatment phases, particularly in procedures involving anesthesia, and that younger children would exhibit higher physiological stress than older ones.

## Materials and Methods

The research was conducted in a pediatric dentistry clinic in northern Italy. The aim was to assess the stress level in children undergoing various DP. A total of 80 children, aged between 5 and 10 years, were treated between January and March 2025.

To carry out this study, specific inclusion and exclusion requirements were established. The inclusion criteria involved pediatric patients aged between 5 and 10 years who had already undergone at least one dental treatment. Participants included underwent specific dental treatments such as Dental Hygiene, Sealants, and Dental Restorations With Anesthesia and Dental Restorations Without Anesthesia.

These four treatments were selected because they represent common and progressively more stimulating procedures in pediatric dentistry, while remaining ethically and clinically noninvasive. Dental hygiene and sealant application are preventive procedures that typically cause minimal discomfort and anxiety. In contrast, restorations—especially those involving local anesthesia—include additional sensory and psychological stimuli, such as injections, drilling sounds, and vibration, which can heighten stress perception. Comparing these procedures, therefore, allows for the evaluation of how different levels of invasiveness, sensory input, and clinical duration influence physiological stress in children under realistic clinical conditions.

Participants were excluded from the study if they were outside the specified age range, suffered from heart disease, or presented other comorbid conditions that could affect the study's integrity, including autism spectrum disorders and other forms of cognitive disability that might impair the ability to cooperate during treatment.

The patients were randomly divided into four groups (20 individuals each). Each group received a different type of dental treatment. Randomization was performed using a computer-generated simple randomization sequence created in Microsoft Excel. Allocation to one of the four treatment groups was carried out by an independent researcher not involved in patient management. The dentist performing the clinical procedures was necessarily aware of the treatment type, while the researcher responsible for collecting and analyzing the HRV data was blinded to treatment allocation to minimize observer bias.

To measure stress levels, a Garmin watch was used. This device measures stress through technology that tracks HRV, providing a score on a scale from 0 to 100, where 0 to 25 indicates low stress, 26 to 50 moderate, 51 to 75 high, and 76 to 100 extremely high stress level. The Garmin stress algorithm is based on HRV analysis using photoplethysmography sensors. Previous validation studies have shown good correlation between the Garmin-derived stress index and standard electrocardiogram-based HRV parameters, such as RMSSD and LF/HF ratio, in both adults and children. Moreover, the system has been successfully applied in pediatric populations to monitor stress and autonomic activity in real-life conditions, supporting its use as a reliable noninvasive tool for research purposes.

As soon as the patient was seated in the dental chair, the watch was placed on their wrist, and stress monitoring began. The monitoring was divided into three distinct phases: before, during, and at the end of each treatment. Before initiating each measurement, individual participant data, including age, sex, and body weight, were entered into the smartwatch user profile. These parameters are required by the device's algorithm to personalize HRV-based stress calculations, as they influence resting heart rate and autonomic balance. All entries were performed and verified by the same trained operator to maintain consistency and reduce measurement variability.

The timing of HRV measurements was standardized and clearly differentiated across the three defined phases. The beginning phase corresponded to the 3-minute period immediately before the start of the clinical intervention, while the child was seated and the instruments were being prepared. The during phase covered the third to sixth minute of active treatment, corresponding to the operative portion of the procedure (e.g., tooth cleaning, drilling, or sealant placement). The end phase referred to the 3-minute period immediately following the completion of the procedure, while the child remained seated in the dental chair. Each phase was operationally marked in the study log by the researcher supervising the session, and the stress values displayed on the smartwatch were manually recorded at approximately 1-minute intervals within each phase, yielding a total of nine standardized measurements per participant.

The study was conducted in accordance with the principles of the Declaration of Helsinki. Ethical approval was waived as no clinical interventions or identifiable personal data were involved, and only physiological measurements were recorded. Written informed consent was obtained from the parents or legal guardians of all participants prior to data collection.

## Statistics

A two-way repeated measures ANOVA was conducted to evaluate the effects of treatment phase (Beginning, During, End) and type of DP (Dental Hygiene, Dental Restorations with Anesthesia, Dental Restorations without Anesthesia, Sealants) on patient-reported stress levels. This approach allowed for the assessment of both within-subject (phase) and between-subject (treatment) factors, as well as their interaction.

Prior to interpretation, the assumption of sphericity was tested using Mauchly's test. When violations were detected, Greenhouse-Geisser and Huynh-Feldt corrections were applied. Effect sizes were calculated using generalized eta squared (ges) to assess the magnitude of observed effects.

Post hoc analyses with Bonferroni correction were performed to explore pairwise comparisons between phases within each treatment group. Additionally, a simple linear regression analysis was conducted to examine the relationship between stress levels and participant age.

All statistical analyses were performed using R software (version 4.3.2), with significance set at *p* < 0.05.

## Results

### Descriptive Analysis: Without Treatment

**Fig. 1:**
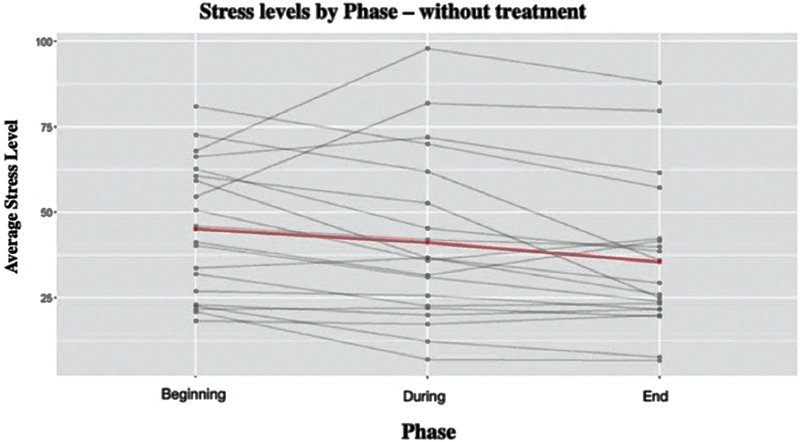
Average stress levels across the three treatment phases (beginning, during, and end) in the control group (without treatment). Each grey line represents an individual participant, while the red line indicates the overall mean trend. A slight decrease in mean stress levels is observed over time, with substantial interindividual variability.

Stress levels recorded without treatment showed a slight overall reduction from the beginning to the end of the observation period. Mean stress values were highest at the beginning (47.9) and lowest at the end (47.9), with a modest peak during the intermediate phase (51.5). Although interindividual variability was high, the general trend indicated a gradual reduction in stress over time. Full descriptive statistics are reported in Table 1 ( Fig. 1 ).

**Table 1:** Descriptive statistics of stress levels (0–100) recorded across the three treatment phases (beginning, during, and end) in the control group

Phase	Mean	Median	Standard deviation	Minimum	Quartile 1	Quartile 3	Maximum
Beginning	47.9	48.3	21.5	14.3	25.6	65.1	94
During	51.5	48.8	25.2	7	25.3	70.4	98
End	47.9	40.8	26.6	6.67	23.8	73	97

Note: Values refer to mean, median, standard deviation, minimum, maximum, and interquartile range (Q1–Q3).

### Descriptive Analysis: Phase and Treatment

**Fig. 2:**
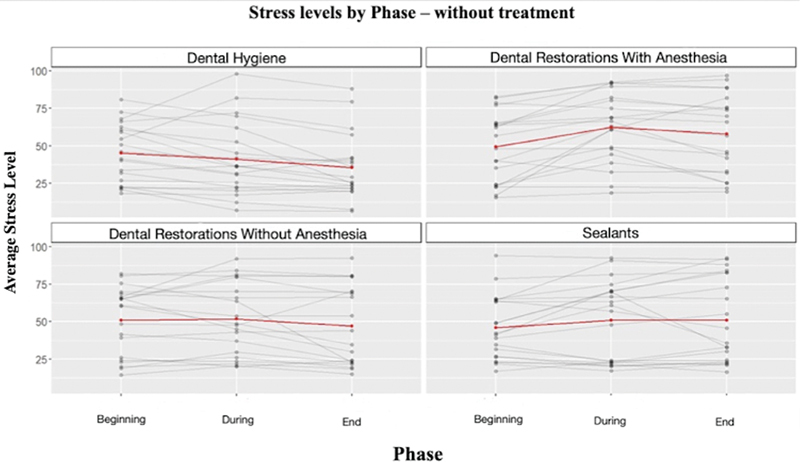
Average stress levels across treatment phases (beginning, during, and end) for each dental procedure. Grey lines represent individual participants; red lines indicate the mean trend within each group. Stress significantly increased during restorations with anesthesia, while a progressive decrease was observed during professional hygiene. No relevant variation occurred in restorations without anesthesia and sealant application.

When stress levels were analyzed according to both treatment and phase, clear differences emerged among the procedures. Dental Hygiene showed a progressive reduction in stress from beginning to end, suggesting that children became more relaxed over time. Dental Restorations With Anesthesia exhibited a significant increase in stress during the procedure, followed by a partial decrease at the end. In contrast, Restorations Without Anesthesia and Sealant applications showed smaller or statistically nonsignificant variations across the three phases. Descriptive statistics for each group and phase are summarized in Table 2 ( Fig. 2 ).

**Table 2:** Results of two-way repeated measures ANOVA assessing the effects of treatment, phase, and their interaction on stress levels

Effect	DFn	DFd	SSn	SSd	F	*p* -Value	ges
Intercept	1	76	578,300	118,033	372	<0.001	0.8135
Treatment	3	76	7,720	118,033	1,657	0.1834	0.0550
Phase	2	152	723	14,514	3,785	0.0249	0.0054
Treatment phase	6	152	2,530	14,514	4,416	0.0004	0.0187
Mauchly's test for sphericity							
Effect	W	*p* -Value					
Phase	0.70	<0.001					
Treatment phase	0.70	<0.001					
Sphericity corrections							
Effect	GGe	p (GG)	HFe	*p* -Value (HF)			
Phase	0.77	0.04	0.78	0.036			
Treatment phase	0.77	0.00	0.78	0.001			

Note: The main effect of phase and the interaction between treatment and phase were statistically significant. Mauchly's test indicated violations of sphericity, for which Greenhouse-Geisser (GGe) and Huynh-Feldt (HFe) corrections were applied.

### ANOVA Results

The results indicated that the main effect of treatment was not statistically significant, suggesting that the different treatments did not significantly affect stress levels when considered in isolation. However, the phase factor showed a significant effect, indicating variations in stress levels over time or across conditions. More importantly, the treatment—phase interaction was highly significant, suggesting that the effect of treatments depends on the phase in which they were applied.

Since the assumption of sphericity was violated (as shown by Mauchly's Test), sphericity corrections were applied (Greenhouse-Geisser and Huynh-Feldt), and even after these corrections, the effects of phase and the interaction remained significant, reinforcing the robustness of the findings ( Table 2 ).

Since the treatment alone was not significantly associated with stress levels, we will now conduct an analysis considering only the phase. In other words, no significant evidence was found linking the treatment to stress levels when evaluated in isolation.

## Post Hoc Test with Bonferroni Correction

Post hoc pairwise comparisons using Bonferroni correction were then performed to further explore these relationships. In the Dental Hygiene group, stress values showed a progressive and statistically significant decrease from the beginning to the end of treatment ( *p* = 0.042), indicating a relaxation effect as the session proceeded. Conversely, in Dental Restorations With Anesthesia, a significant increase in stress was observed during the active phase of treatment ( *p* = 0.001), reflecting heightened anxiety or discomfort during the procedure. The Dental Restorations Without Anesthesia and Sealant groups did not display statistically significant differences between phases, suggesting a more stable stress response across the treatment duration.

These findings collectively confirm that children's stress levels are not static but fluctuate during dental treatment, depending on both the type of intervention and its phase. The data emphasize that procedures involving anesthesia tend to elicit higher stress during active phases, whereas preventive procedures such as hygiene appear to promote stress reduction over time. Complete statistical results, including ANOVA tables and post hoc comparisons, are reported in Table 3 .

**Table 3:** Post hoc pairwise comparisons (Bonferroni-corrected) of mean stress levels between treatment phases (beginning, during, end) for each dental procedure

Treatment	Phase 1	Phase 2	Mean phase 1	Mean phase 2	*p* -Value
Dental hygiene	During	Beginning	41.1500	45.1500	0.5707
	End	Beginning	35.5167	45.1500	0.0420
		During	35.5167	41.1500	0.0414
Dental restorations with anesthesia	During	Beginning	62.3833	49.4333	0.0011
	End	Beginning	57.9167	49.4333	0.1624
		During	57.9167	62.3833	0.2108
Dental restorations without anesthesia	During	Beginning	51.7167	50.8167	1.0000
	End	Beginning	46.9667	50.8167	1.0000
		During	46.9667	51.7167	0.2599
Sealants	During	Beginning	50.9167	46.0167	0.2668
	End	Beginning	51.0667	46.0167	0.5203
		During	51.0667	50.9167	1.0000

Note: Significant differences were observed in Dental Hygiene and Restorations with Anesthesia, indicating phase-dependent variations in stress response.

## Discussion

Several studies have identified stress in pediatric dentistry as a significant issue. Stress not only poses difficulties during treatment but may also persist into adolescence, potentially leading to avoidance of dental care or challenging behavior. To prevent this detrimental cycle, it is essential to identify stressed children as early as possible. Early identification allows for the implementation of appropriate pediatric management techniques aimed at controlling stress response before it becomes more deeply ingrained. 19 20 Similar to our findings, a study conducted in children analyzed the variability in stress across different phases of dental treatment using salivary cortisol as a biomarker. Saliva samples were collected from a total of 39 children aged 2 to 5 years at three time points: upon waking, upon arrival at the dental office , and 25 minutes after the DP, and were tested for cortisol to measure stress levels. 21

Another comparable study, also using salivary cortisol as a biomarker, reached the same conclusion, supporting the idea that stress levels in children vary significantly during different phases of dental treatment. Salivary cortisol levels were measured in eight children (mean age: 5.6 years) undergoing their initial dental treatment at four stages: prior to treatment, during cavitation, during liner placement, and during restoration. 22

However, a previous study measured stress by salivary cortisol as a biomarker in children aged 4 to 5 years; their analysis revealed no statistically significant differences in salivary cortisol levels prior to treatment, postoral prophylaxis, or postfluoride treatment at the first and second appointments of both groups. At the first appointment, the fluoride treatment caused a significant increase in the salivary cortisol level over the pretreatment level in both groups, but it was not evident in either of the two groups studied at the second appointment. 23 Although the study did not find significant differences in salivary cortisol levels during the different types of treatment, the results may be attributed to the small sample size.

The results of this study highlight that stress levels in children undergoing dental treatments are not constant but vary depending on the type of procedure. A progressive decrease in stress was observed during Dental Hygiene, suggesting that children may adapt to this procedure as it progresses. In contrast, Dental Restorations With Anesthesia led to a significant increase in stress during the procedure itself, indicating that the procedure involving anesthesia caused a peak in stress during its execution. Dental Restorations Without Anesthesia showed minimal and statistically nonsignificant variations in stress levels, and the same was observed for Sealants, where no meaningful differences were recorded between treatment phases. These findings suggest that the type of treatment directly influences the trajectory of stress, with some treatments causing more stress during execution, while others promote progressive relief.

The higher stress levels observed during restorations with anesthesia may be explained by a combination of physiological and psychological factors. The anticipation of an injection, the sound and vibration of the handpiece, and the perception of numbness are well-known triggers of dental anxiety in children. Physiologically, local anesthesia can transiently activate the SNS, increasing heart rate and reducing HRV, particularly in younger or first-time patients. Psychologically, children often interpret anesthesia as a signal that “something painful is coming,” which amplifies anticipatory stress.

One study evaluated twenty healthy children aged 4 to 8 years undergoing their first dental visit and requiring both a restoration and an extraction.

Notably, cavity preparation (restoration) was identified as the most stressful phase, than other treatments. 24

Similar patterns of procedure-specific stress responses have been reported in a separate study involving 50 healthy patients with a mean age of approximately 34 years. The results demonstrated that stress levels varied depending on the type of treatment. Cortisol levels decreased by approximately 15% from baseline to the end of the procedure in patients undergoing an examination, root canal therapy, or restorative treatment. In contrast, cortisol levels were elevated at the end of the procedure in the prophylaxis (55%) and extraction (148%) groups compared with baseline values. The authors concluded that procedures such as dental examinations, restorative treatments, and root canal therapy were associated with low stress levels, whereas tooth extraction and prophylaxis resulted in noticeably higher stress levels. 25

A systematic review aimed to determine whether root canal treatment (RCT) causes greater physiological or psychological stress than other DP. To achieve this, the authors conducted a comprehensive search of peer-reviewed literature published in English between January 1990 and January 2023. After screening a total of 3,639 records, they selected 23 studies that met all inclusion criteria. However, the included studies were generally of low methodological quality, with the overall certainty of evidence rated as low to very low. When comparing RCT to other treatments, such as extractions, the evidence remained inconclusive. 26

The results of this study indicate that age has a statistically significant influence on stress levels in children undergoing dental treatments. As people get older, their stress tends to decrease. However, age accounts for only a small proportion of the overall variability in stress responses, explaining approximately 5.3% of the observed differences.

Similar results were observed in a study involving 3,200 children aged 4 to 6 and 9 to 11. This study examined the impact of general fears, parental dental anxiety, and socio-economic factors on children's dental fear levels. The findings indicated that dental fear decreases with age and is positively correlated with general fears and maternal dental anxiety, supporting the conclusion that these factors play a significant role in influencing children's dental fear. 27

In alignment with these observations, another study aimed to assess the psychological impact of dental interventions on children, focusing on anxiety and stress levels as well as coping mechanisms. Two groups were compared: children with orthodontic problems and those with general dental issues. The results revealed moderate anxiety and stress levels across both groups, with Sarason's anxiety scale scores averaging 20.63 ± 8.37 for the dental group and 18.66 ± 6.85 for the orthodontic group. Stress test scores were similarly close, at 7.63 ± 3.45 and 7.76 ± 3.78, respectively. Statistical analysis using one-way ANOVA identified significant differences in anxiety and stress scores related to age and gender, confirming that these demographic factors influence the psychological response to dental treatment. However, no significant difference was found between the two groups in terms of stress or anxiety, indicating that the type of dental condition itself does not substantially affect these outcomes. These findings reinforce the importance of considering age-related differences when assessing and managing dental anxiety and stress in pediatric patients. Understanding that stress and anxiety tend to decline with age can guide the development of age-appropriate interventions and coping strategies to improve the dental experience for children. 28

These results align with our own findings, which revealed a significant role of age in stress perception during dental treatment. Although our initial hypothesis posited no statistically significant difference in stress levels across age groups, our data demonstrated otherwise. Taken together with the findings of Rantavuori et al, this suggests that age is a relevant factor in pediatric dental stress and should be considered when developing age-appropriate strategies for anxiety management in clinical practice.

In contrast to our study, a previous study aimed to present normative data on dental fear in the Dutch child population by identifying not only highly fearful children but also those at risk of developing high dental fear. This was done using the Dutch parent-reported version of the Children's Fear Survey Schedule, Dental Subscale (CFSS-DS). The researchers calculated total CFSS-DS scores across different age groups and separately for boys and girls. Their analysis found no significant effects of age on dental fear in either the general or referred samples. Instead, significant effects were observed for gender, with girls exhibiting higher fear levels, and for cultural background, with children from non-Western backgrounds scoring higher. These findings align with previous research that has reported mixed results regarding how dental fear changes with age. While some studies suggest a slight decrease in fear as children grow older, this trend is often weak or nonlinear. Overall, the study concluded that age alone is not a strong predictor of dental fear, and that any age-related changes likely reflect developmental differences in how fear is expressed and managed, rather than a genuine reduction in anxiety. 29 Although age is commonly assumed to influence dental fear in children, this study did not find a significant relationship between age and fear levels. This absence of an age effect may be explained by the fact that fear does not necessarily decrease with age, but rather changes in its expression. As children mature, they may learn to manage or conceal their fear, resulting in less observable anxious behavior, even though the internal experience of fear remains. Moreover, factors such as cultural background, temperament, and individual psychological traits may exert a stronger influence on dental fear than chronological age alone. These findings underscore the importance of considering a range of personal and contextual variables when assessing fear responses in pediatric dental patients.

From a clinical perspective, these findings highlight several important implications. First, wearable devices that monitor HRV can serve as objective tools to identify moments of peak stress in real time, enabling pediatric dentists to adjust communication tone, pause briefly, or use distraction techniques when stress rises. Second, for procedures known to elicit greater anxiety—such as restorations with anesthesia—preappointment desensitization, tell-show-do approaches, and computer-controlled anesthesia systems may help minimize fear and physiological arousal. Finally, integrating stress monitoring into behavioral management protocols could support individualized treatment planning, fostering better cooperation, reducing the need for pharmacological sedation, and enhancing the overall patient experience.

## Conclusion

This study confirms that stress levels in pediatric patients undergoing DP vary significantly across treatment phases and are influenced by the type of procedure. In particular, professional dental hygiene was associated with a significant reduction in stress over time, while dental restorations with anesthesia caused a marked increase in stress during the procedure. Restorations without anesthesia and sealants showed no significant variations, indicating more stable stress responses. The use of smart watches for real-time HRV monitoring proved to be a practical and noninvasive tool for assessing physiological stress in children during dental care. Additionally, age was found to be inversely associated with stress levels, although it explained only a small portion of the overall variability. These findings highlight the importance of tailoring behavioral and clinical strategies to the child's age and the type of procedure, and they support the integration of wearable devices in pediatric dentistry for early identification and management of stress.
